# Ramucirumab in second‑line advanced colorectal cancer therapy: A study on therapeutic outcomes and hepatic sinusoidal platelet aggregation

**DOI:** 10.3892/ol.2024.14572

**Published:** 2024-07-15

**Authors:** Keisuke Kimura, Yosuke Katata, Yuzo Umeda, Takehiro Tanaka, Shuya Yano, Kazuhiro Yoshida, Toshiyoshi Fujiwara, Yoshiko Mori, Takeshi Yamada, Takeshi Nagasaka

**Affiliations:** 1 Department of Gastroenterological Surgery, Okayama University Hospital, Okayama 700-8558, Japan; 2 Department of Clinical Oncology, Kawasaki Medical School Hospital, Kurashiki, Okayama 701-0192, Japan; 3 Department of Pathology, Okayama University Graduate School of Medicine, Dentistry and Pharmaceutical Sciences, Okayama 700-8558, Japan; 4 Department of Clinical Genetics, Saitama Medical Center, Saitama Medical University, Kawagoe, Saitama 350-8550, Japan; 5 Department of Digestive Tract and General Surgery, Saitama Medical Center, Saitama Medical University, Kawagoe, Saitama 350-8550, Japan; 6 Department of Gastrointestinal and Hepato-Biliary-Pancreatic Surgery, Nippon Medical School, Tokyo 113-8602, Japan; 7 Division of Advanced Oncology, Kawasaki Medical School, Kurashiki, Okayama 701-0192, Japan

**Keywords:** advanced colorectal cancer, ramucirumab, liver metastases, platelet aggregation

## Abstract

The present study investigated the role of ramucirumab (RAM) in treating liver metastases (LMs) as a second-line or salvage treatment in patients with advanced CRC. Of the 36 patients, 21 (58%) received RAM plus folinic acid, fluorouracil and irinotecan (FOLFIRI) as second-line treatment, whereas 15 (42%) received it in a salvage setting. The median overall survival time was 23 months [95% confidence interval (CI), 12–34 months] for those in the second-line treatment group and 8 months (95% CI, 5–19 months) for those in the salvage treatment group. Of the 36 patients, 14 (39%) underwent surgical resection of LMs during chemotherapy. A total of 6 patients underwent surgical resection for LMs for the first time during second-line RAM plus FOLFIRI (RAM-LM); of the remaining 8 patients, 6 underwent resection of LMs during first-line bevacizumab (BEV)-based chemotherapy (BEV-LM). Immunohistochemical analysis of CD42b showed that the platelet aggregation score (CD42b score), which ranges from 0 (absence of deposition) to 3 (presence of linear deposition), tended to decrease with the increasing duration of treatment with both RAM and BEV. Although there was no significant difference in the mean duration of anti-VEGF antibody treatment between the BEV-LM and RAM-LM groups, the median CD42b score was higher in the RAM-LM group (median CD42b score, 3; range, 0–3) compared with that in the BEV-LM group (median CD42b score, 1; range, 0–3; P=0.01), suggesting that RAM induces a different degree of platelet aggregation in liver sinusoids compared to BEV.

## Introduction

Colorectal cancer (CRC) is a global health concern, being the third most diagnosed type of cancer and the second leading cause of cancer-related deaths worldwide, with 1,926,118 new cases and 903,859 deaths reported in 2022 (1). The treatment of advanced CRC, particularly when accompanied by liver metastases (LMs), which affect ~50% of patients with CRC during the course of the disease, presents a substantial challenge worldwide (2). Notably, there has been a shift towards novel treatment strategies, with ramucirumab (RAM), a human IgG1 monoclonal antibody that targets the extracellular domain of VEGF receptor (VEGFR)2, attracting considerable attention as a potential treatment for various malignancies, including CRC (3).

The therapeutic efficacy of RAM has been extensively examined in patients with advanced CRC and other types of cancer, and has shown promising results (4–6). Notably, in a randomized, double-blind, multicenter phase III study, RAM in combination with folinic acid, fluorouracil and irinotecan (FOLFIRI) demonstrated a median overall survival (OS) time of 13.3 months and a median progression-free survival (PFS) time of 5.7 months when used as second-line treatment, suggesting that this may be a promising option for patients with metastatic CRC (mCRC) (4).

Current research has also assessed the effects of RAM on LMs derived from CRC, with evidence suggesting that RAM is more favorable for patients without liver-only metastases (4). Notably, unlike other anti-VEGF antibodies, RAM is effective against advanced hepatocellular carcinoma (5); however, the understanding of the benefits patients at different disease stages could obtain from treatment with RAM, and the exact mechanisms through which RAM affects the pathology, especially of the liver, remains limited.

Addressing these issues, the present study aimed to assess the potential role of RAM in second-line or salvage treatments in patients with advanced CRC and LMs. This study focused on a specific patient population, those with advanced CRC and LMs receiving RAM and FOLFIRI as second-line or salvage treatments, and aimed to evaluate the effectiveness of this treatment strategy based on PFS, OS, overall response rate (ORR) and LM resection rate, parameters that have yet to be adequately investigated in previous studies involving RAM (7,8). In addition, the present study aimed to elucidate the impact of RAM on the pathology of LMs, specifically on platelet aggregation in liver sinusoids. This study may contribute to the literature, and has the potential to influence future research and clinical practice in the treatment of patients with advanced CRC.

## Materials and methods

### Patients

The present retrospective cohort study included 36 patients who received RAM plus FOLFIRI treatment for unresectable CRC as second-line or salvage chemotherapy. The study was conducted between September 15, 2016, and May 5, 2019, at the Kawasaki Medical School Hospital (Kurashiki, Japan) and Okayama University Hospital (Okayama, Japan). Relevant clinical data, including patient demographics, laboratory parameters, imaging findings, treatment toxicities and prognostic outcomes, were retrospectively collected.

### Treatment

The RAM plus FOLFIRI regimen was administered intravenously every 2 weeks. RAM was administered at a dose of 8 mg/kg over 1 h on day 1, folinic acid was administered at a dose of 400 mg/m^2^ over 2 h on day 1, irinotecan was administered at 150 mg/m^2^ over 150 min on day 1 of each cycle; fluorouracil was administered intravenously as a bolus of 400 mg/m^2^ on day 1, followed by continuous infusion of fluorouracil at a dosage of 2,400 mg/m^2^ over 46 h on days 1–3. Dose adjustments or interruptions were made in accordance with the institutional clinical practice guidelines. Treatment response was evaluated using the Response Evaluation Criteria in Solid Tumors (RECIST) version 1.1 (9). Regular imaging evaluations, predominantly using computed tomography, were performed every 2 months, corresponding to once every four treatment cycles, as per standard institutional practice.

### Calculation of the duration of bevacizumab (BEV) administration

The patients were divided into two treatment groups: The second-line group consisted of 58% (21 patients) of the patients and included patients who received RAM plus FOLFIRI as a second-line treatment, whereas the salvage-line group consisted of 42% (15 patients) of the patients, who were treated with RAM plus FOLFIRI as salvage therapy (third-line or later). The duration of BEV administration was calculated before the administration of RAM, excluding the BEV withdrawal period. In the second-line group, among 21 patients, 4 did not use BEV in the first-line treatment, whereas the remaining 17 received BEV plus oxaliplatin with a fluoropyrimidine (5-FU or capecitabine). In the salvage-line group, the duration of BEV was calculated from the treatment history. Among the 15 patients in the salvage-line group, 13 were treated with BEV plus oxaliplatin with a fluoropyrimidine (5-FU, capecitabine or S1) as first-line therapy. Of the remaining 2 patients, 1 patient received BEV plus irinotecan with capecitabine; the other received panitumumab plus oxaliplatin with 5-FU. BEV was used in 8 of the 15 patients in the second-line treatment and was administered again in 3 patients in the third-line or later chemotherapy before RAM administration.

### Criteria for hepatic resection of LMs

The criteria for hepatic resection were as follows: i) LMs must be considered surgically resectable (regardless of the number of LMs); ii) the disease must be controlled by chemotherapy [partial response (PR) to stable disease (SD) for ≥2 months]; iii) the only other organ with metastasis, other than the liver, was the lungs.

### Immunohistochemistry for CD42b

Formalin-fixed paraffin-embedded tissue specimens were used for immunohistochemical (IHC) staining. Serial 3-µm tissue sections were prepared and subjected to either hematoxylin and eosin (H&E) staining or IHC staining using mouse monoclonal anti-CD42b (cat. no. NBP1-28457; clone MM2/174; Novocastra; Leica Biosystems). Immunostaining was performed using an automated Bond-III Stainer (Leica Microsystems GmbH) according to the manufacturer's instructions.

Briefly, tissue specimens were fixed in 10% neutral-buffered formalin at room temperature for 24–48 h. The sections were first dewaxed with BOND Dewax Solution, after which, antigen retrieval was performed by heating the tissue sections in BOND Epitope Retrieval ER1 Solution (citrate-based buffer, pH 6.0) at 95–100°C for 20 min. Endogenous peroxidase activity was quenched using the Refine Detection Kit Peroxide Block (Leica Biosystems) for 5 min at room temperature and blocking was performed using normal goat serum (cat. no. 5425; Cell Signaling Technology, Inc.) at room temperature for 20 min. Subsequently, the sections were incubated with anti-CD42b (1:100) at room temperature for 30 min. Secondary detection was performed using the Refine Detection Kit Polymer (cat. no. DS9800; Leica Biosystems) at room temperature for 10 min. Chromogen detection was carried out using the Refine Detection Kit Mixed DAB Refine (Leica Biosystems), according to the manufacturer's instructions. For H&E staining, the sections were automatically stained using the Bond-III Stainer with 0.1% hematoxylin for 5 min at room temperature, followed by 0.5% eosin staining for 2 min at room temperature.

Images were captured using a light microscope (Olympus BX53; Olympus Corporation) with a 40× objective lens. CD42b immunostaining results were categorized into four stages based on the observed staining pattern: Score 0, absence of deposition; score 1, presence of fine granular deposition; score 2, presence of coarse granular deposition; and score 3, presence of linear deposition.

### Statistical analysis

Statistical analyses were performed using JMP Pro 17 (SAS Institute, Inc.). PFS was defined as the time from the initiation of RAM plus FOLFIRI treatment to disease progression. OS was defined as the time from the initiation of RAM plus FOLFIRI treatment to death. Cumulative survival probabilities were estimated using the Kaplan-Meier method. The ORR was calculated as the proportion of patients achieving a complete response (CR) or PR, and the disease control rate (DCR) was defined as the proportion of patients achieving CR, PR or SD. In cases where the RECIST evaluation differed from the attending physician's assessment, the latter's judgment was included in the evaluation. Differences in CD42b scores, presented as median (IQR), and the mean duration of anti-VEGF antibody treatment before liver resection between the groups were evaluated using a Mann-Whitney U test. The correlation between the CD42b score and the duration of anti-VEGF antibody treatment (months) before liver resection was examined using logistic regression analysis with Spearman's coefficient (ρ). Categorical variables were examined by Fisher's exact test. P<0.05 was considered to indicate a statistically significant difference.

## Results

### Patients and clinical responses

This study evaluated 36 patients with mCRC who were treated with RAM plus FOLFIRI. The characteristics of the patients enrolled in the present study are shown in Table I. Of these, 58% were female. With respect to primary tumor location, 31% of primary tumors were on the right side. Additionally, 67% presented with LMs and 94% had an Eastern Cooperative Oncology Group (ECOG) performance status (PS) of 0–1 (10). Moreover, 64% of the patients had a mutant RAS status.

In the second-line group, the median age was 57 years (range, 16–73 years) and 57% of patients were female. All patients in this group had an ECOG PS of 0–1. Of the patients in this group, 29% had right-sided primary tumors located from the cecum to the transverse colon, 62% presented with LMs and 67% had a mutant RAS status. In the salvage group, the median age was 63 years (range, 37–76 years) and 60% were female. Among these patients, 87% had an ECOG PS of 0–1. Additionally, 33% had right-sided primary colon tumors, 60% had a mutant RAS status and 73% had LMs.

The present study also reviewed the duration of BEV treatment prior to RAM administration, categorizing it into the following: No use, <6 months, 6–12 months, and ≥13 months. Across the entire cohort, 4 patients had not been treated with BEV prior to RAM, 9 were treated for <6 months, 7 for 6–12 months and 16 for ≥13 months. In the second-line group, 4 patients had no prior BEV treatment, 6 were treated for <6 months, 6 for 6–12 months and 5 for ≥13 months. In the salvage group, all had received prior BEV treatment, with 3 treated for <6 months, 1 for 6–12 months and 11 for ≥13 months.

On average, patients underwent 7.4 cycles of RAM plus FOLFIRI treatment [95% confidence interval (CI), 5.3–9.5]. Those in the second-line group averaged 8.2 cycles (95% CI, 5.0–11.4), whereas the salvage group averaged 6.2 cycles (95% CI, 3.7–8.7). The number of chemotherapy lines after RAM administration was not significantly different between the second-line and salvage groups for 0–2 lines (excluding cases with unknown data), although there were slightly different percentages of patients undergoing 1 or 2 lines of chemotherapy in each group. Notably, only patients in the second-line group had ≥3 chemotherapy lines.

Finally, clinical responses were assessed. Neither the second-line nor salvage treatment groups exhibited CR. The ORR was 10% in the second-line group and 7% in the salvage group. The DCR was notably higher in the second-line group at 81% compared to 47% in the salvage group. In the second-line group, 71% of patients displayed SD, whereas in the salvage group, this was observed in 40%. PD was observed in 14% of the second-line group and 53% of the salvage group.

### PFS and OS

**Figure 1.**
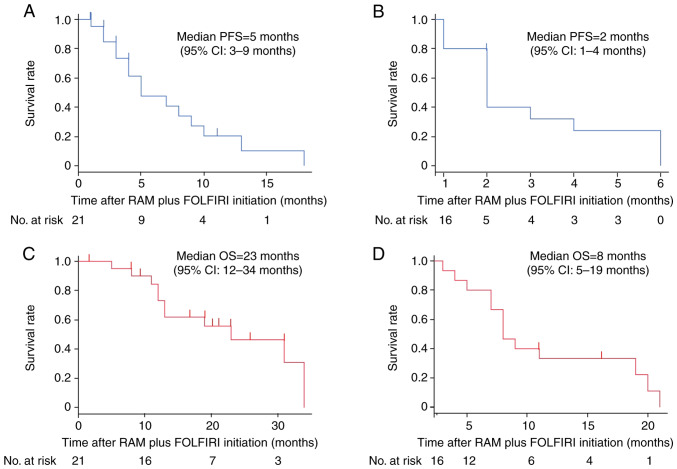
PFS and OS. Kaplan-Meier estimates depicting the PFS rate in the (A) second-line group and (B) salvage group, as well as the OS rate in the (C) second-line group and (D) salvage group. CI, confidence interval; FOLFIRI, folinic acid, fluorouracil and irinotecan; OS, overall survival; PFS, progression-free survival; RAM, ramucirumab.

PFS and OS were evaluated following the initiation of RAM plus FOLFIRI treatment. For the second-line group, the median PFS was 5 months (95% CI, 3–9 months; Fig. 1A). Conversely, the median PFS in the salvage group was shorter, at 2 months (95% CI, 1–4 months; Fig. 1C). The median OS in the second-line group was 23 months (95% CI, 12–34 months; Fig. 1B), which is in contrast to the salvage group, where the median OS was 8 months (95% CI, 5–19 months; Fig. 1D).

### Characteristics of patients undergoing hepatic resection for LMs

**Figure 2.**
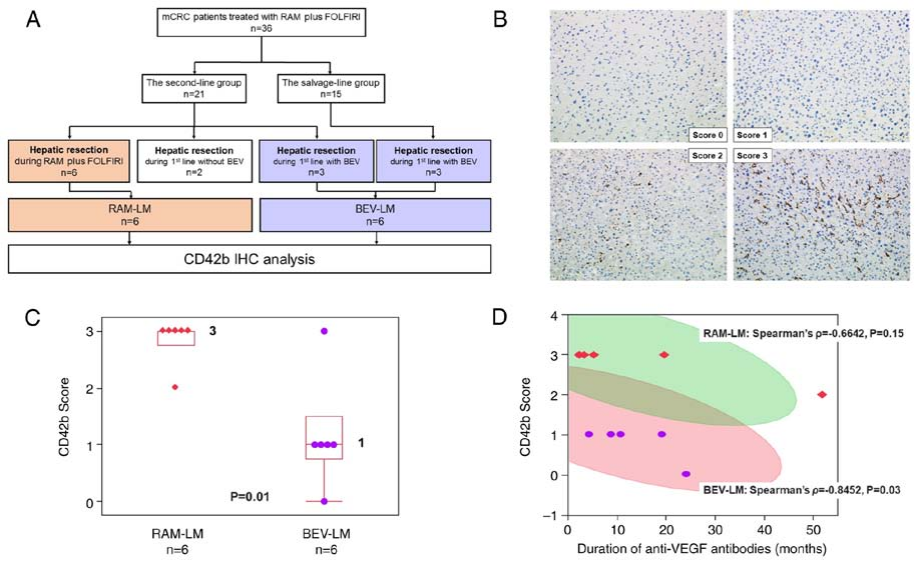
Features of patients undergoing hepatic resection for LMs. (A) Study flow chart. (B) Examples of CD42 IHC staining and scoring. Images show examples of CD42 staining with corresponding scores: 0 (absence of deposition), 1 (presence of fine granular deposition), 2 (presence of coarse granular deposition) and 3 (presence of linear deposition). Magnification, ×400. (C) CD42 score distribution. Each box represents a quartile range, and whiskers represent the maximum and minimum values. The number to the right of each box represents the median. The P-value was calculated using a Mann-Whitney U test. (D) Correlation between CD42 score and the duration of anti-VEGF antibody treatment. Each red square represents a case in the RAM-LM group, while each purple dot represents a case in the BEV-LM group. The green-shaded area denotes the 95% CI for the scatterplot matrix of the RAM-LM group. Similarly, the red-shaded area represents the 95% CI for the scatterplot matrix of the BEV-LM group. BEV, bevacizumab; CI, confidence interval; FOLFIRI, folinic acid, fluorouracil and irinotecan; IHC, immunohistochemical; LM, liver metastasis; mCRC, metastatic colorectal cancer; RAM, ramucirumab.

Of the 36 patients, 14 (39%) underwent surgical resection of LMs during chemotherapy. In this subset, 6 patients underwent their first surgical resection for LMs during second-line RAM plus FOLFIRI treatment, forming the RAM-LM group. Of the remaining 8 patients, 2 underwent their first resection during first-line chemotherapy before BEV initiation and thus these cases were excluded, and 6 underwent their first resection during BEV-based first-line chemotherapy, constituting the BEV-LM group (Fig. 2A). The details of patients who underwent LM resection can be found in Table II. The RAM-LM group showed a trend toward a shorter duration of BEV administration, except in one case; this was due to the early failure of front-line BEV plus oxaliplatin-based chemotherapy. Notably, 1 patient did not receive BEV but was treated with RAM plus FOLFIRI as a second-line regimen because of relapse during oxaliplatin-based adjuvant chemotherapy.

Since H&E staining showed no hepatic sinusoid injury in any LM samples resected after BEV treatment or those resected after BEV followed by RAM administration (data not shown), the association between anti-VEGF antibody exposure duration and platelet aggregation (CD42b) score was subsequently assessed. While CD42b score in the liver sinusoids of the BEV-LM group was influenced by the duration of BEV treatment only, 1 of the 6 patients in the RAM-LM group was BEV-naive (CD42b score=3), while the remaining 5 patients received BEV prior to RAM; therefore, the CD42b score of the RAM-LM group was influenced by the duration of both BEV and RAM treatment. Although there was no significant difference in the mean duration of anti-VEGF antibody treatment between the BEV-LM and RAM-LM groups (Table II), the RAM-LM group had a notably higher median platelet aggregation (CD42b) score (median value, 3; range, 0–3) compared with the BEV-LM group (median value, 1; range, 0–3; P=0.01; Fig. 2B and C). Additionally, a tendency for a negative correlation between the duration of anti-VEGF antibody exposure and the CD42b score was detected in both the BEV-LM and RAM-LM groups (Fig. 2D).

## Discussion

The present study provides a comprehensive exploration of the therapeutic outcomes in relation to the administration of RAM and FOLFIRI in patients with mCRC. Most patients in the present cohort had RAS mutations, reflecting the frequency of this mutation in CRC (11). Additionally, right-sided primary tumors were frequently observed in the present cohort, a characteristic associated with poorer prognosis in CRC (12).

Despite the modest ORR observed in the present study, this aligned with the results of previous studies assessing the efficacy of RAM and FOLFIRI combination therapy in patients with mCRC (4,13). Notably, the DCR was markedly higher in the group receiving second-line treatment, implying a potentially superior efficacy of RAM plus FOLFIRI as second-line therapy. Conversely, disease progression was observed more frequently in the salvage group, suggesting a possible decline in treatment efficacy in the advanced therapy stages.

The present findings regarding median PFS and OS were congruent with the outcomes reported in the RAISE study, a phase III trial examining RAM plus FOLFIRI in patients with mCRC (4). The relatively lower survival rates of the salvage group in the present study highlight the inherent challenges of treating mCRC in its advanced stages; this predicament has been well-documented in other studies (13,14).

The present study further explored the pivotal role of platelets in tumor angiogenesis, growth and metastasis, and assessed the interactions between anti-VEGF antibodies, platelet aggregation and the liver microenvironment in patients with mCRC. Notably, the selective binding of RAM to VEGFR2 inhibits its ligand interaction, thus blocking the angiogenesis pathway (15). This mechanism is distinct from BEV, which sequesters VEGF-A itself, preventing the interaction between VEGF-A, VEGFR1 and VEGFR2 on the cell surface (16). Building on the current understanding, the present study was initiated by examining the pathological characteristics of LMs resected after the administration of BEV and those following RAM administration. Initially, H&E staining showed no hepatic sinusoid injury in any LM samples resected after BEV treatment or those resected after BEV followed by RAM administration. As a result, the focus was shifted to platelet behavior, which could not be discerned by H&E staining. Notably, the CD42b score tended to decrease as the duration of anti-VEGF antibody administration increased in both the BEV-LM group and the RAM-LM group. In addition, it was indicated that the mean CD42b score was significantly higher in the RAM group than that in the BEV group, despite no difference in the mean duration of anti-VEGF antibody administration between the two groups. Although the number of cases was small, among the second-line group, 13 patients had LMs, of which 6 patients underwent hepatic resection. This observation led to the hypothesis that platelet aggregation within hepatic sinusoids, observed in patients who underwent resection of LMs after RAM plus FOLFIRI, might have an antitumor effect on LMs. This finding suggests that upfront use of RAM could enhance the resection rate of LMs in patients with mCRC, potentially offering a new therapeutic strategy for these patients, especially those with RAS mutations (4).

In the context of hepatic injury associated with chemotherapy for mCRC treatment, oxaliplatin-induced hepatic sinusoidal injury (HSI) is a recognized condition. Oxaliplatin-induced HSI reveals specific pathological changes, including edema of hepatic sinusoidal epithelial cells, expansion of the intercellular space and ongoing disintegration of the hepatic sinusoid wall, leading to necrosis, detachment of the sinus endothelium, and sinus expansion and rupture during the early and middle stages of HSI (17). Although the precise mechanism underlying this toxicity remains unclear, it appears that oxaliplatin-induced reactive oxygen species and the subsequent increase in VEGF-A levels may serve a critical role (18,19). In rat models of HSI induced by monocrotaline, a pyrrolizidine alkaloid, VEGF inhibition with either sorafenib or regorafenib protected against this injury (20,21).

In contrast, the association between platelet aggregation and HSI is unclear. Given the known mitigating effect of BEV on HSI (22), the present study aimed to investigate the correlation between the duration of anti-VEGF antibody treatment and CD42b score. A negative correlation was observed between the duration of anti-VEGF antibody exposure and the CD42b score in both the BEV-LM and RAM-LM groups. Pathological examination using H&E staining revealed no markers of oxaliplatin-induced HSI, despite both groups receiving oxaliplatin regimens. In addition, the RAM-LM group, treated with irinotecan following first-line oxaliplatin-based chemotherapy, showed no signs of irinotecan-induced fatty liver. This suggests that RAM itself may have an effect on the increasing CD42b scores, i.e., by promoting platelet aggregation.

It is widely recognized that patients with RAS-mutant CRC lack effective targeted therapy, as anti-EGFR antibodies have demonstrated limited efficacy in this subtype (23). Therefore, the present findings underscore the potential of RAM as a promising therapeutic strategy for such cases. However, it is essential to note that these interpretations, although promising, remain largely speculative at this stage, and necessitate further investigation for their validation and potential clinical application.

The present study is not without limitations. The modest sample size could have influenced both the statistical power and the broader applicability of the results. Given the retrospective design of the present study, there is an inherent risk of selection and information biases. Additionally, the effect of RAM plus FOLFIRI on quality of life, a pivotal factor when considering treatment options for advanced CRC, was not evaluated. Moreover, the present data did not encompass potential molecular markers beyond RAS and BRAF, which might have a role in determining the response to RAM plus FOLFIRI.

Nevertheless, the results of the present study indicated that the unique pattern of intrasinusoidal platelet aggregation observed could act as a potential biomarker, signaling a favorable response to RAM treatment. This hypothesis might be attributed to the specific mode of action of RAM related to VEGFR2. While these insights are encouraging, they remain conjectural, and require further scrutiny and confirmation.

Future research must address these shortcomings, evaluating the precise mechanisms underlying hepatic sinusoidal platelet aggregation in patients receiving anti-VEGF antibodies, and comprehensively investigating its prospects as a predictive biomarker for treatment outcomes. Such endeavors may corroborate the present conclusions and provide pivotal insights into the broader context of advanced CRC diagnosis and management.

## Figures and Tables

**Table I.** Patient characteristics and clinical responses.

Characteristic	Total (n=36)	Second-line treatment group (n=21)	Salvage treatment group (n=15)
Median age, years (range)	58 (16–76)	57 (16–73)	63 (37–76)
Female sex, n (%)	21 (58%)	12 (57%)	9 (60%)
Right primary tumor location, n (%)	11 (31%)	6 (29%)	5 (33%)
With liver metastases, n (%)	24 (67%)	13 (62%)	11 (73%)
ECOG PS 0–1, n (%)	34 (94%)	21 (100%)	13 (87%)
RAS mutant, n (%)	23 (64%)	14 (67%)	9 (60%)
BRAF mutant, n (%)	1 (3%)	0 (0)	1 (7%)
MSI-high, n (%)	0 (0)	0 (0)	0 (0)
Mean no. of cycles of RAM plus	7.4 (5.3–9.5)	8.2 (5.0–11.4)	6.2 (3.7–8.7)
FOLFIRI (95% CI)			
Duration of BEV treatment before			
RAM induction, n (%)			
No use	4 (11%)	4 (19%)	0 (0)
<6 months	9 (25%)	6 (29%)	3 (20%)
6 to 12 months	7 (19%)	6 (29%)	1 (7%)
≥13 months	16 (44%)	5 (24%)	11 (73%)
No. of chemotherapeutic lines after			
RAM therapy, n (%)			
0	11 (31%)	6 (29%)	5 (33%)
1	13 (36%)	6 (29%)	7 (47%)
2	5 (14%)	2 (10%)	3 (20%)
3 or more	4 (11%)	4 (19%)	0 (0)
Unknown	3 (8%)	3 (14%)	0 (0)
Response, n (%)			
Complete response	0 (0)	0 (0)	0 (0)
Partial response	3 (8%)	2 (10%)	1 (7%)
Stable disease	21 (58%)	15 (71%)	6 (40%)
Progressive disease	11 (31%)	3 (14%)	8 (53%)
Not evaluated	1 (3%)	1 (5%)	0
Overall response rate, n (%)	3 (8%)	2 (10%)	1 (7%)
Disease control rate, n (%)	24 (66%)	17 (81%)	7 (47%)

BEV, bevacizumab; ECOG PS, Eastern Cooperative Oncology Group performance status; FOLFIRI, folinic acid, fluorouracil and irinotecan; MSI, microsatellite instability; RAM, ramucirumab.

**Table II.** Detailed information of patients undergoing hepatic resection for LMs.

A, BEV-LM										

Sex	Age, years	Primary tumor location	Stage at diagnosis	Number of LMs at resection	Surgical procedure	CD42b score	Duration of anti-VEGF treatment, months			

							BEV	RAM	Total	Mean (range)
M	66	Sigmoid colon	IV	4	Anatomical subsegmentectomy (S5) and lateral segmentectomy (S2+3)	1	10.5	-	10.5	11.3 (2–24)^a^
F	70	Ascending colon	IV	3	Posterior segmentectomy (S6+7)	1	4	-	4	
F	76	Sigmoid colon	IV	3	Right lobectomy (S5+6+7+8)	1	19	-	19	
F	70	Ascending colon	IV	2	Partial resection	1	8.5	-	8.5	
M	67	Rectum	II	1	Partial resection	0	24	-	24	
F	62	Ascending colon	IV	9	Anatomical subsegmentectomy (S8) and partial resection ×2	3	2	-	2	

**B, RAM-LM**										
**Sex**	**Age, years**	**Primary tumor location**	**Stage at diagnosis**	**Number of LMs at resection**	**Surgical procedure**	**CD42b score**	**Duration of anti-VEGF treatment, months**			

							**BEV**	**RAM**	**Total**	**Mean (range)**

M	58	Transverse colon	pIIIa	8	Anatomical segmentectomy (S3+4+8) and partial resection ×4	3	2.5	2.5	5	14.4 (2–52)^a^
F	52	Ascending colon	pIIIb	6	Anatomical subsegmentectomy (S5) and partial resection ×2	3	0	2	2	
F	71	Cecum	pIIIa	1	Anterior segmentectomy (S5+8)	3	1.5	1.5	3	
M	58	Sigmoid colon	IV	20	Anterior segmentectomy (S5+8) + partial resection ×3	3	13	6.5	19.5	
F	38	Sigmoid colon	IV	2	Left lobectomy (S2+3+4)	3	4	1	5	
F	62	Rectum	IV	8	Anatomical subsegmentectomy (S2) and partial resection ×5	2	40	12	52	

a BEV-LM vs. RAM-LM, P=0.81. P-value was evaluated using a Mann-Whitney U test. BEV, bevacizumab; LM, liver metastasis; RAM, ramucirumab.

## Data Availability

The data generated in the present study may be requested from the corresponding author.
